# *Chromobacterium violaceum* infection on lower limb skin: A case report

**DOI:** 10.1097/MD.0000000000024696

**Published:** 2021-02-12

**Authors:** Peng Zhang, Jie Li, Yan-zhen Zhang, Xiao-ning Li

**Affiliations:** Department of Clinical Laboratory, Yijishan Hospital of Wannan Medical College, Wuhu, Anhui, China.

**Keywords:** *Chromobacterium violaceum*, mass spectrum analysis, skin infection

## Abstract

**Rational:**

*Chromobacterium violaceum* is a motile gram-negative bacterium. This bacterium commonly grows in tropical or subtropical areas in sewage and can cause opportunistic infections.

**Patient concerns:**

A 50-year-old Chinese man had a skin ulcer in the middle of his left leg in front of the tibia. The diameter of the wound was 3.0 cm, the exudation was obvious, and necrotic tissue was attached to the wound. One week previously, he was working in a field where he accidentally punctured his left leg.

**Diagnosis:**

*C violaceum* infection was diagnosed as per the results of pathogen culture from the infection site.

**Interventions:**

He was treated with piperacillin/tazobactam (3.375 g/12 h iv) and levofloxacin (0.5 g/24 h iv) for 5 days.

**Outcomes:**

The patient showed good response to therapy and was discharged on day 18 after wound healing.

**Lessons:**

*C violaceum* rarely infects humans. When an infection is suspected, samples should be immediately sent for microbial culture. Timely treatment on the basis of drug sensitivity test results can prevent further complications.

## 1 Introduction

*Chromobacterium violaceum* is a gram-negative, facultative anaerobic, and opportunistic pathogen that grows in the water and soil of tropical and subtropical regions.^[1]^*C violaceum* can grow on nutrient agar and are observed as smooth and low protuberant colonies with a metallic dark purple luster. *C violaceum* is rarely reported to cause human disease, and only about 150 cases have been reported to date since the first report of a case in Malaysia in 1927.^[2]^ However, in recent years, more reports are being published about this infection. Thus far, most reports have been published in southern Asia and South American regions associated with skin injury, sepsis,^[3]^ and fatal liver abscess with a mortality rate of up to 50%.^[4]^ To understand the clinical features and drug resistance of *C violaceum* infection, we report the clinical features, bacterial identification, and drug susceptibility test results of a patient with *C violaceum* skin infection who was admitted to our hospital. The study was approved by the Institutional Review Board of Yijishan Hospital of Wannan Medical College (Protocol No.: WF2020021).

## 2 Case report

A 50-year-old man suffered a puncture on his left leg in an accident when he was doing farm work in a field, the local skin was broken, ulcerated, and bleeding. At that time, the patient did not seek medical treatment and the wound eventually scabbed over. About 1 week later, when the patient was working in the field again, the wound opened and the local skin turned black and necrotic. The patient visited our hospital for treatment on September 2, 2020. The wound was located in the middle of the left leg just in front of the tibia. The diameter of the wound was 3.0 cm, the epidermis was damaged, the base was dirty, exudation was obvious, and necrotic tissue was attached to the wound. He was diagnosed with skin infection, ulceration, and necrosis of the middle and front of the left leg. The wound surface was cleaned routinely when the relevant examinations were performed. The patient's electrocardiography and chest computed tomography results were normal. Clinical laboratory tests showed normal routine blood parameters and biochemical indicators. The patient was then treated with cefotiam (2.0 g/12 h iv) and hydrocortisone (100 mg/24 h iv) for 3 days; however, he did not respond well to treatment. Considering a possible infection, we collected purulent secretion from the ulcerated wound for microbial culture and identification.

**Figure 1:**
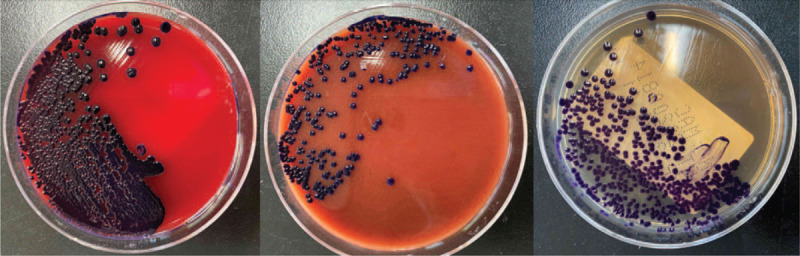
*C violaceum* isolated from the skin wound of a 50-year-old Chinese man who accidentally punctured his left leg, growing on blood agar (left), chocolate (middle), and Maconkey agar (right) after incubation at 37 °C for 24 hours. *C violaceum* = *Chromobacterium violaceum*.

**Figure 2:**
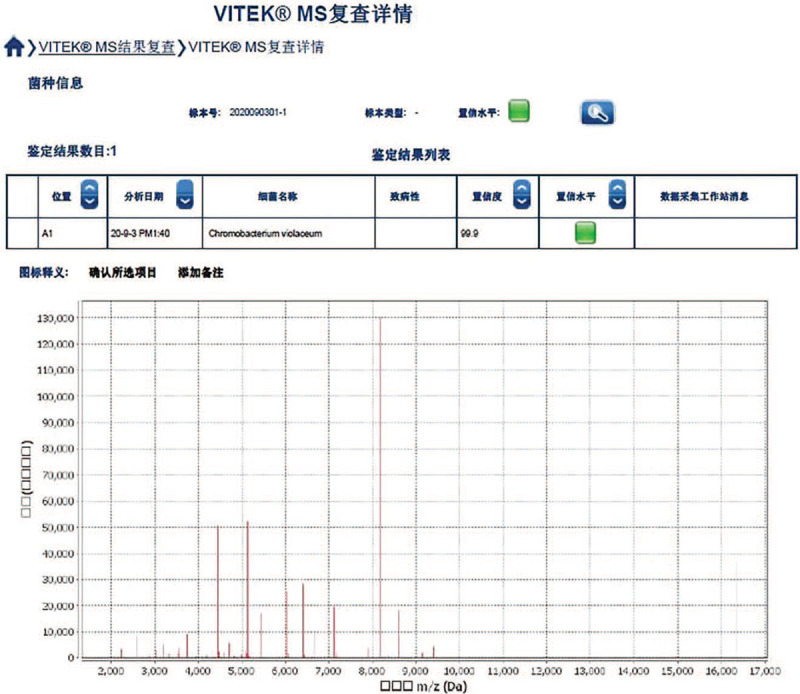
*C violaceum* was identified using VITEK MS (bioMérieux SA, Marcy l’Etoile, France). *C violaceum* = *Chromobacterium violaceum*.

**Figure 3:**
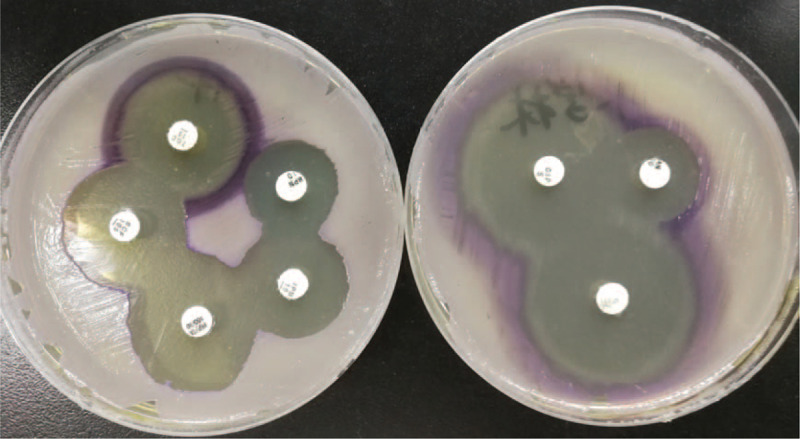
Results of drug susceptibility test using the Kirby–Bauer method.

The sample was subjected to Gram staining and subculturing. Gram staining revealed gram-negative rods of about 0.6–0.9 × 1.5–3.0 μm with bipolar staining. Upon subculturing, colonies showed outstanding violet and black pigmentation (Fig. 1) on blood, chocolate, and Maconkey agar plates. The culture was identified as *C violaceum* using VITEK 2 compact automated system and VITEK MS (bioMérieux SA, Marcy l’Etoile, France) (Fig. 2). Drug susceptibility was tested using Kirby–Bauer method, and the results interpreted as per CLSI M45-A3 showed sensitivity to piperacillin/tazobactam, cefperazone/sulbactam, tigecycline, meropenem, imipenem, levofloxacin, ciprofloxacin, and amikacin (Fig. 3). Prompt combination treatment with piperacillin/tazobactam (3.375 g/12 h iv) and levofloxacin (0.5 g/24 h iv) was administered for 5 days. Eventually, the ulcer surface on the left leg shrank, and new granulation tissue grew well. There was no redness and swelling around the wound. The secretion was sent for culture again, and the results showed no *C violaceum* growth. The patient recovered and was discharged after 1 week of continued medication.

## 3 Discussion

*C violaceum* is a gram-negative bacterium widely found in the water and soil. It is a common opportunistic pathogen that rarely infects humans; however, it can cause urinary tract infection, diarrhea, local abscesses, multiple organ abscesses, and sepsis when the patient's resistance is low.^[5,6]^ More cases have recently been reported in South America and southern Asia following its first report in humans in 1927.^[7]^ Owing to the limited information on its biological and clinical characteristics, patients infected with *C violaceum* often have an acute onset and rapid progression of the infection. *C violaceum* can rapidly invade the blood and organs from the infected site, if not treated promptly and effectively, patients become prone to complications such as sepsis and even death.^[8]^

In our case, the patient did not take the initial infection seriously. When the wound opened again, the infection spread rapidly in the local skin. The secretion from the infected site was sent for bacterial culture and identification. Purple colonies that grew on solid medium fermented glucose, trehalose, N-acetylglucosamine, and gluconate but not L-arabinose and D-galactose. The bacterium was finally identified as *C violaceum* using VITEK 2 compact automated system and VITEK MS with biochemical reactions and protein peak spectrum.

First, the patient was administered empirical antimicrobial therapy with cefotiam, however, this was ineffective. To our knowledge, no guidelines currently exist for the interpretation of the antimicrobial sensitivity test data for *C violaceum*, most likely owing to their rarity in clinical settings.^[9]^*C violaceum* is highly resistant to ampicillin, penicillin, and cephalosporin but sensitive to fluoroquinolones, aminoglycosides, and carbapenems. In our study, the organism isolated from the skin infection was sensitive to piperacillin/tazobactam, cefperazone/sulbactam, tigecycline, meropenem, imipenem, levofloxacin, ciprofloxacin, and amikacin. In other studies, the susceptibility of *C violaceum* to the tested antibiotics was similar to that found in our study.^[10]^ Clinicians in our study chose piperacillin/tazobactam and levofloxacin for treatment. There was obvious improvement in the local infection of the patient. One week thereafter, the secretion was sent for culture again, and the results showed no *C violaceum* growth.

Early diagnosis is challenging and the infection progresses rapidly, therefore, patients often develop toxic shock and multiple organ dysfunction syndrome before the laboratory results are obtained. In case of suspected *C violaceum* infection, the secretion and blood of the patient should be sent for bacterial culture and drug sensitivity testing as early as possible to enable a definite diagnosis and select the appropriate antibiotics for timely and appropriate treatment.

## Acknowledgments

The authors thank Liwen Bianji, Edanz Editing China (www.liwenbianji.cn/ac), for editing a draft of this manuscript.

## Author contributions

**Data curation:** Peng Zhang, Jie Li.

**Formal analysis:** Peng Zhang, Xiao-ning Li.

**Investigation:** Jie Li, Yanzhen Zhang.

**Methodology:** Peng Zhang, Jie Li.

**Resources:** Jie Li, Yan-zhen Zhang.

**Supervision:** Xiaoning Li.

**Validation:** Peng Zhang, Yanzhen Zhang, Xiaoning Li.

**Writing – original draft:** Peng Zhang.

**Writing – review & editing:** Peng Zhang.

## Abbreviations

Abbreviation: C violaceum = Chromobacterium violaceum.
